# Supplementary material for: The interactome of intact mitochondria by cross-linking mass spectrometry provides evidence for coexisting respiratory supercomplexes
Source: Mol Cell Proteomics. 2017 Dec 8;17(2):216–32. doi: 10.1074/mcp.RA117.000470 (PMC5795388; doi:10.1074/mcp.RA117.000470)
Supplement: Supplemental Data [file supp_RA117.000470_133922_0_supp_23978_fzffwf.zip › spectra_annotation/mito_DR_spectra_annotation/306-1-5-1-10-1.pdf]

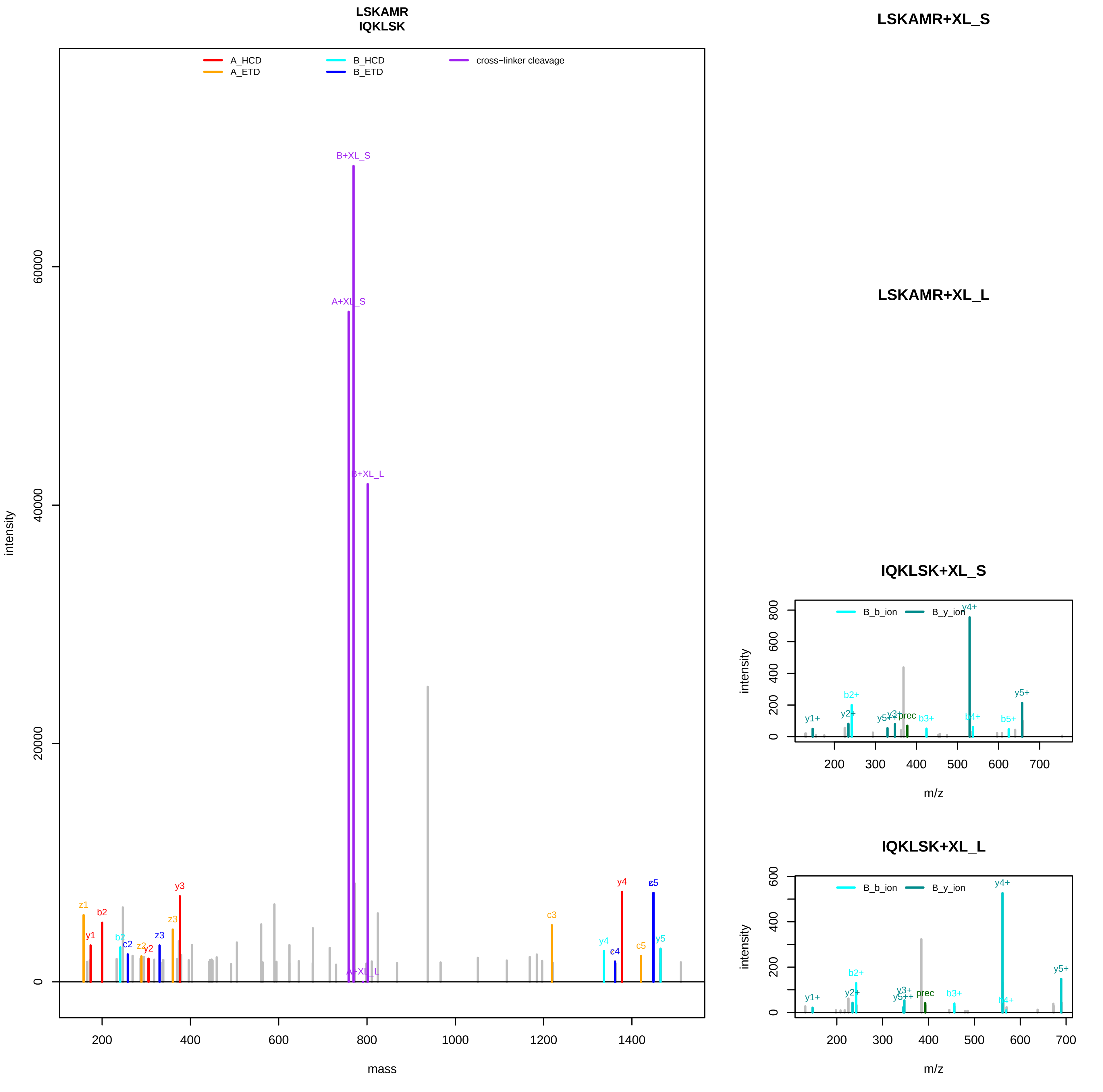
LSKAMR
IQKLSK
A_HCD
A_ETD
B_HCD
B_ETD
cross−linker cleavage
B+XL_S
A+XL_S
B+XL_L
A+XL_L
z1
y1
b2
b2
c2
z2
y2
z3
z3
y3
c3
y4
c4
c5
z5
y4
y5
intensity
mass
0
20000
40000
60000
200
400
600
800
1000
1200
1400
LSKAMR+XL_S
LSKAMR+XL_L
IQKLSK+XL_S
B_b_ion
B_y_ion
y1+
y2+
b2+
y5++
y3+
prec
b3+
y4+
b4+
b5+
y5+
intensity
m/z
0
200
400
600
800
300
500
700
IQKLSK+XL_L
B_b_ion
B_y_ion
y1+
y2+
b2+
y5++
y3+
prec
b3+
y4+
b4+
y5+
intensity
m/z
0
200
400
600
300
500
700
